# Spatial variability of soil attributes and risk of phosphorus loss in different soil classes of the Potengi River Basin, Brazil

**DOI:** 10.1007/s10661-026-15402-1

**Published:** 2026-05-05

**Authors:** José Arthur do Nascimento Ramalho, Karina Patrícia Vieira da Cunha, Matheus Natan Ferreira Alves de Sousa, Caio Victor Macêdo Pereira, Lara Fernandes de Medeiros, Carlos Wilmer Costa

**Affiliations:** Federal University of Rio Grande Do Norte, Natal, Brazil

**Keywords:** Soil science, Soil mapping, Geoprocessing, Semiarid, Principal component analysis, Two-way cluster

## Abstract

The spatial variability of soil attributes plays an important role in hydrological processes, soil fertility, and environmental conservation in tropical semiarid regions. Texture, organic matter (OM), and available phosphorus (P) directly influence nutrient dynamics and the risk of phosphorus export to water bodies. This study analyzes the spatial distribution of soil texture, OM, and P in different soil classes of the Potengi River Basin (PRB), assessing their relationship with weathering and phosphorus mobility. A total of 110 soil samples were collected from different pedological classes, following standardized physical and chemical analysis methods. The data were spatially interpolated using the IDW method in ArcMap 10.8, and statistical analyses, including correlation, principal component analysis (PCA), and two-way cluster analysis, were applied to identify distribution patterns. The results revealed a predominance of sandy soils, moderate OM levels, and high phosphorus content. PCA identified two soil groups: Group 1, composed of more developed soils with higher clay and OM content, and Group 2, consisting of less developed soils with a higher risk of phosphorus export. The negative correlation between P and clay content emphasized the influence of texture on nutrient retention and mobility. This study highlights the relevance of spatial analyses for soil quality assessment and provides essential insights for sustainable land management strategies aimed at mitigating diffuse phosphorus pollution in semiarid watersheds.

## Introduction

Soils play a critical role in sustaining terrestrial ecosystems, modulating the hydrologic cycle, and influencing water and nutrient dynamics, thereby supporting agricultural productivity. Additionally, they provide essential ecosystem services, including carbon storage and regulation of nutrient fluxes for vegetation (Weil & Brady, 2016). Soil texture, a key physical attribute, exerts a profound impact on these processes by controlling drainage, water-holding capacity, and nutrient availability for plant growth (Pelegrino et al. 2025) .

Soil texture, characterized by the proportions of sand, silt, and clay, profoundly impacts biological, physicochemical, and hydrological processes within the soil. Sandy soils, exhibiting lower weathering intensity, display high permeability and low water retention capacity, rendering them more susceptible to nutrient leaching and erosion. Conversely, more developed clayey soils possess higher water and nutrient retention capacities but may impede infiltration and water movement through the profile (de Souza et al., 2018). Furthermore, soil mineralogy plays a pivotal role in carbon sequestration and nutrient sorption, directly influencing their availability to plants and retention within the soil (Pelegrino et al. 2025).

Among the essential chemical constituents of soil, organic matter (OM) and available phosphorus (P) are paramount. The presence of OM in soil is vital for maintaining its structure and fertility, as it helps retain moisture, sequester carbon, and release nutrients, making it a key factor in soil quality (Araújo Filho et al., 2022; Prezotti & Guarçoni, 2013). However, inadequate management practices can accelerate OM degradation, intensify carbon mineralization, and enhance greenhouse gas emissions (GHG), leading to disruptions in biogeochemical cycles and compromising the sustainability of agroecosystems (Shuite et al. 2025). Phosphorus, an essential macronutrient for agricultural productivity, exhibits limited mobility in tropical soils, rendering its availability highly sensitive to soil physicochemical characteristics and management practices. Excessive phosphorus in soil, resulting from unregulated fertilizer application and leaching, can exacerbate eutrophication processes in water bodies, particularly in tropical semiarid regions (Bonilla et al. 2023; Cunha & Cunha, 2023; Rocha Júnior et al. 2024).

In semiarid regions, such as northeastern Brazil, the inherent fragility of soils is accentuated by escalating anthropogenic pressures, intensifying processes like structural degradation, fertility decline, and erosion vulnerability (Cunha & Cunha, 2023; Sousa et al., 2024). Intensive land use, particularly for agricultural, industrial, and urban purposes, can compromise soil functionality, impairing water infiltration, nutrient retention, and productivity, potentially leading to desertification and jeopardizing water resources (Castro & dos Santos, 2020; Costa et al., 2024). Furthermore, inadequate soil conservation practices and inefficient fertility management exacerbate environmental degradation, amplifying GHG emissions and depleting soil carbon reserves (Shuite et al. 2025).

In this context, assessing soil quality at the watershed scale is crucial for environmental planning, particularly in semiarid regions, where water scarcity and vulnerability to cyanobacteria blooms due to phosphorus contamination of reservoirs pose significant threats to water quality for supply (Rocha Júnior et al. 2024; Bonilla et al. 2023). The spatial distribution of soil texture fractions, OM, and P provides invaluable insights into pedogenetic processes, enabling an understanding of the interrelationships between these attributes, such as the impact of soil weathering intensity on their characteristics. This information facilitates the identification of areas with higher potential for phosphorus export to aquatic systems.

The Potengi River Basin (PRB) exemplifies the environmental challenges inherent to semiarid regions. Due to its significant socioeconomic and environmental importance, the PRB supports a diverse range of human activities, including agriculture, livestock, industry, mining, and urbanization, which can substantially alter its natural characteristics (Pereira et al., 2024). These anthropogenic interventions impact hydrological processes, enhancing erosion and degrading soil and water quality (Pereira et al., 2024; Sousa et al., 2024). Therefore, understanding the soil properties in this region is essential for informing sustainable management practices.

In light of these considerations, this study aims to investigate the spatial distribution of clay, silt, sand, available phosphorus, and organic matter contents across different soil classes within a predominantly semiarid tropical watershed in northeastern Brazil. Furthermore, this research seeks to assess how these attributes reflect the degree of weathering and influence the potential for phosphorus export to water bodies, consequently contributing to the exacerbation of eutrophication processes. Based on these findings, this study intends to elucidate the local edaphic dynamics and provide insights for sustainable basin management, ultimately contributing to environmental conservation and informing strategies for improved land use and land cover in the region.

## Materials and methods

### Study area

**Fig. 1:**
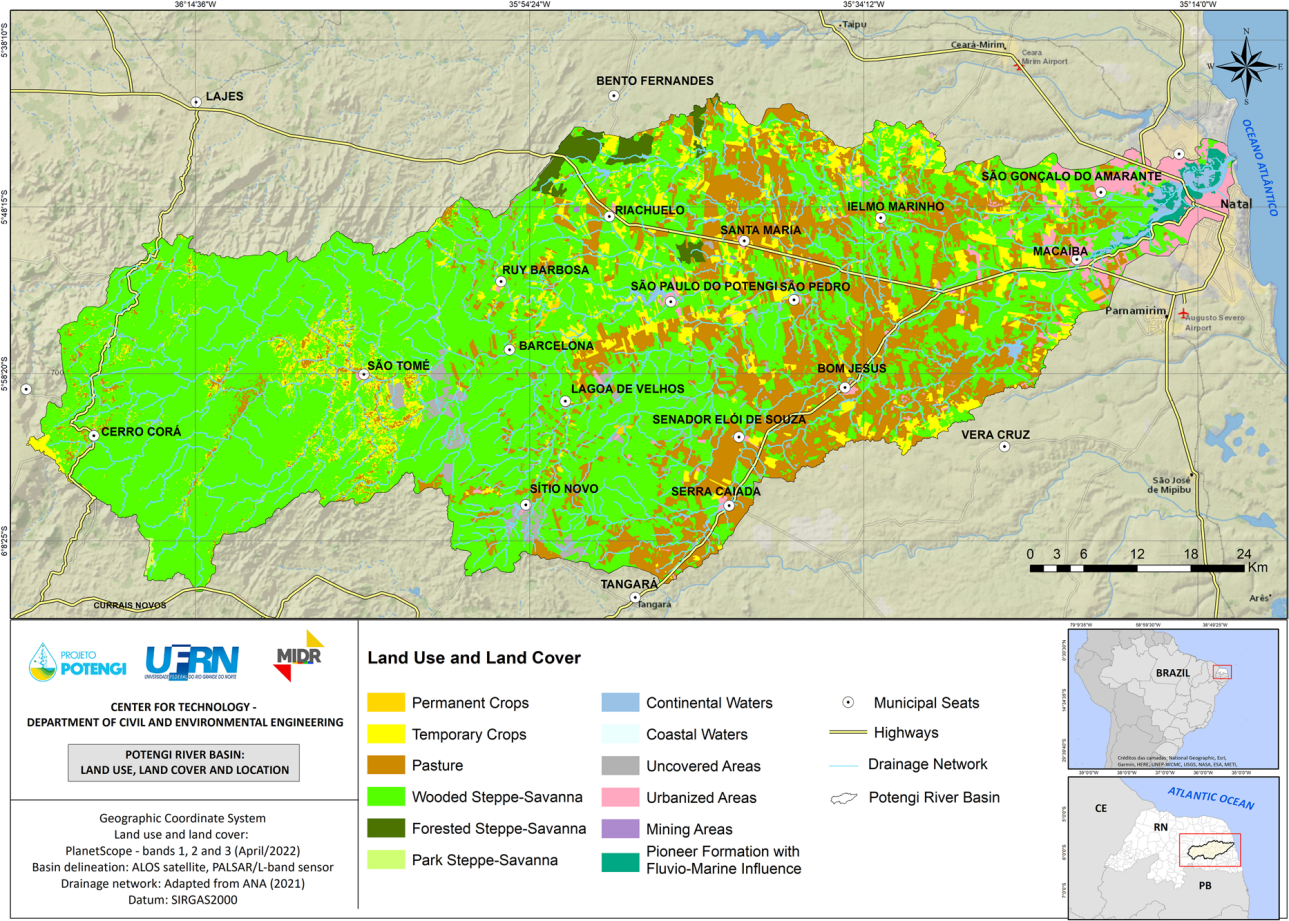
Location map with land use and land cover of the Potengi River Basin. Source: authors (2025)

The Potengi River Basin is situated within the Eastern Northeast Atlantic Hydrographic Region of Brazil and is entirely located in the state of Rio Grande do Norte (RN), covering an area of approximately 4093 km^2^, which accounts for approximately 7.7% of the state’s total area (Fig. 1). The PRB is distinguished by its significant socioeconomic and environmental importance, with a drainage network comprising 427 rivers and encompassing 25 municipalities. The basin’s main watercourse, the Potengi River, originates in a sedimentary environment in the central mountains of Cerro Corá municipality and flows approximately 135 km from west to east, ultimately discharging into the Atlantic Ocean in the state capital, Natal (Miranda & Farias, 2021; Sousa et al., 2024). The basin stretches approximately 50 km in the north–south direction and is bounded by the Ceará-Mirim and Doce River basins to the north, the Trairi and Pirangi basins to the south, the Atlantic Ocean to the east, and the Piranhas-Açu basin to the west (Souza & Amorim, 2022).

The Potengi River, whose name is derived from the term “Rio Grande” and also lends its name to the state, plays a vital role in supporting various economic activities, including fishing, aquaculture, shrimp farming, mining, agriculture, and livestock watering. However, the river is currently in a state of vulnerability, as its natural characteristics have been significantly altered by chemical, physical, and biotic modifications resulting from anthropogenic interventions and erosion processes intensified by land use and land cover (Pereira et al., 2024; Sousa et al., 2024; Torres et al., 2019). These changes may compromise the quality of water resources, exacerbate environmental degradation, and undermine the sustainable development of the region.

The land use and land cover map reveals that 2.74% of the basin area is urbanized, with a focus on the Greater Natal region to the east. Pastures occupy 20.81% of the area, predominantly located in the central-eastern region, while areas designated for agriculture, including both temporary and permanent crops, account for 8.45%. Mining activities, although present, represent only 0.03% of the total area. Consequently, 32.03% of the basin has been anthropized, whereas the remaining 67.97% is comprised of water bodies and native vegetation, primarily characterized by wooded steppe savannas in the central and western portions of the basin (Pereira et al., 2024; Sousa et al., 2024).

**Fig. 2:**
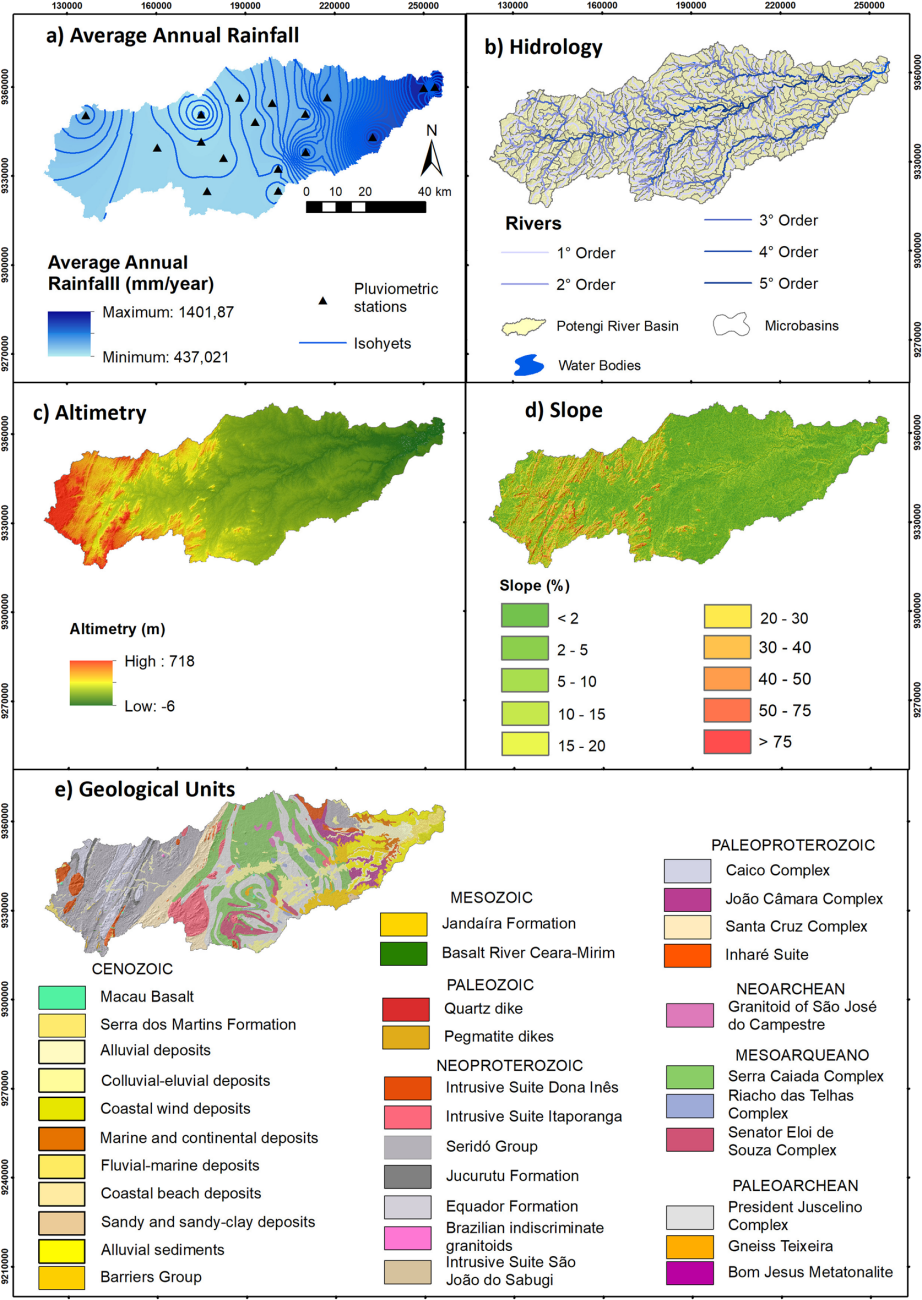
Table of maps characterizing the study area. Rainfall (**a**) Hidrology (**b**). Altimetry (**c**). Slope (**d**). Geological Units (**e**). Source: authors (2025)

The climate of the PRB is characterized by three distinct types, according to Köppen’s classification. In the far western part of the basin, the BSw‛h’ climate predominates, classified as very hot and semiarid, with a late rainy season occurring in the fall. In the central-western region, the BSs‛h’ climate is observed, also semiarid and very hot, but with a slightly earlier onset of the rainy season. In these semiarid portions of the PRB, the average annual rainfall is limited to around 500 mm (Fig. 2). In contrast, the eastern portion of the basin is characterized by the As’ climate, which is tropical and rainy with dry summers and concentrated precipitation during the fall (Barros et al., 2013), with an average annual precipitation of up to 1400 mm.

These climatic conditions exert a direct influence on hydrological processes, resulting in intermittent and ephemeral rivers in the semiarid region and perennial rivers in the tropical rainy portion. The western region is characterized by higher elevations, reaching up to 718 m, and steeper slopes, whereas the eastern region is flat and near sea level. From a geological perspective, pre-Cambrian crystalline geological units predominate in the middle and upper reaches of the river, while Cenozoic sedimentary deposits are concentrated in the lower course. Notably, a sedimentary cap is present in the area of the main springs, as reported by the Brazilian Geological Service (SGB 2012, 2013, 2014a and 2014b).

**Fig. 3:**
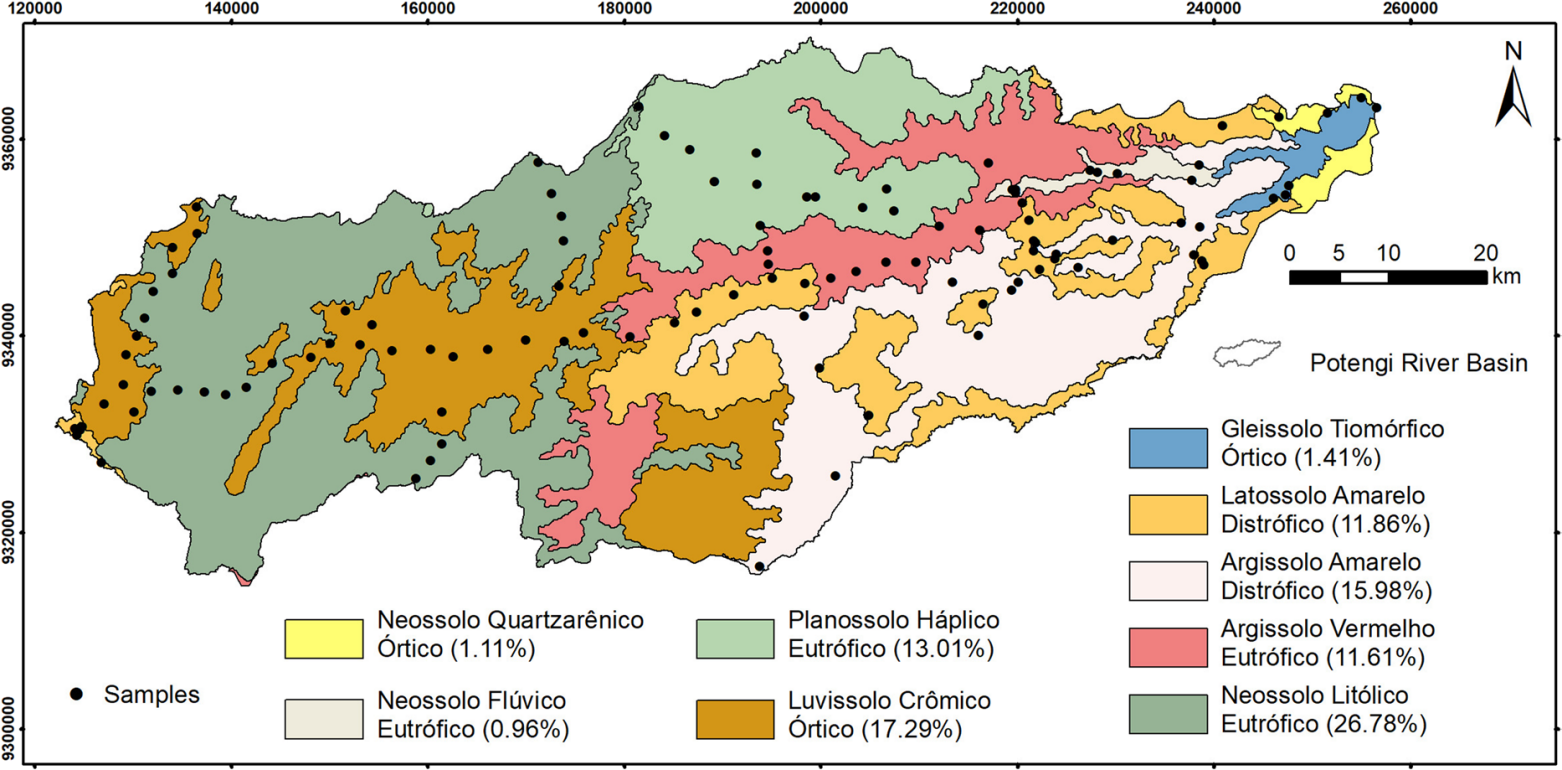
Pedological units and sampling points in the Potengi River Basin. Source: authors (2025)

The pedological units of the study area (Fig. 3), based on the RADAMBRASIL surveys (Brasil, 1981), reveal the presence of nine distinct soil classes according to the Brazilian Soil Classification System (Santos et al., 2018) and International Union of Soil Sciences (IUSS, 2022) in parentheses. The western and central-southern regions are predominantly characterized by Luvissolo Crômico Órtico (Luvisols), whereas the Borborema Plateau, located in the western region, is primarily covered by Neossolo Litólico Eutrófico (Leptosols). A homogeneous presence of Planossolo Háplico Eutrófico (Planosols) is observed in the northern-central area. In contrast, the central and eastern regions of the basin exhibit a mosaic of Argissolo Vermelho Eutrófico (Acrisols), Argissolo Amarelo Distrófico (Acrisols), and Latossolo Amarelo Distrófico (Ferralsols). Near the mouth of the Potengi River, Neossolo Flúvico Eutrófico (Fluvisols), Neossolo Quartzarênico Órtico (Arenosols), and Gleissolo Tiomórfico Órtico (Gleysols) are present. The pedological diversity of the basin has a direct impact on its water retention capacity, fertility, and susceptibility to erosion, which are crucial factors for planning soil use and conservation in the region.

The high level of anthropic occupation, coupled with intensive land use for agriculture and livestock farming, exerts significant pressure on the natural resources of the basin. Deforestation, soil compaction, and improper fertilizer application contribute to soil degradation, excessive nutrient loading into water bodies, and the intensification of eutrophication processes, ultimately compromising the environmental quality of the region.

### Experimental design

The experimental design was structured to ensure spatial representativeness and precision in soil analyses within the PRB. To achieve this, a total of 110 sampling points were strategically distributed across the basin, taking into account the pedological variability of the study area and accessibility via highways and local roads. Each sampling point was georeferenced using the Geocentric Reference System for the Americas (SIRGAS2000), zone 25S, and the UTM projection system. An information plan was created to facilitate subsequent spatial interpolation of the data in a Geographic Information Systems (GIS) environment. Therefore, clinical trial number is not applicable.

Soil samples were collected following a standardized protocol, wherein composite samples were formed by combining five simple samples collected at a depth of 0–20 cm. Upon collection, the samples were stored in sealed plastic bags, properly labeled and identified, and maintained at room temperature until laboratory analysis.

In the laboratory, the samples underwent a standardized preparation procedure, involving oven drying, crushing, and sieving through a 2-mm mesh, yielding the Fine Oven-Dried Soil fraction, as described by Teixeira et al. (2017). The physical and chemical characterization of the soil followed established protocols (Teixeira et al., 2017): Granulometry was determined using the pipette method, enabling the quantification of sand, silt, and clay fractions. Available phosphorus was quantified by colorimetry after extraction with Mehlich-1 solution. Total organic carbon (TOC) was determined using the modified Walkley–Black method and subsequently converted to organic matter using a conversion factor of 1.724, assuming a humus carbon content of approximately 58% (Silva et al., 1999).

Spatial patterns of soil attributes were evaluated using inverse distance weighting (IDW) interpolation in ArcMap 10.8. Although sampling was randomly distributed, access limitations resulted in an irregular spatial configuration with heterogeneous point density. Under these conditions, ordinary kriging produced unstable variogram models and high uncertainty, preventing its reliable application. Therefore, IDW was adopted as a deterministic approach suitable for exploratory spatial analysis. The resulting maps should be interpreted as qualitative representations of spatial trends rather than quantitative predictions.

The analyses were performed using data from 110 soil samples collected across the PRB. Principal component analysis (PCA) and two-way cluster analysis were applied to identify and group the factors that most strongly differentiate soil classes in terms of their potential susceptibility to phosphorus contribution within the basin. Pearson’s correlation coefficient (*r*) was used to evaluate the relationships among physical and chemical soil attributes. All statistical analyses and graphical outputs were conducted using RStudio software.

## Results and discussion

**Fig. 4:**
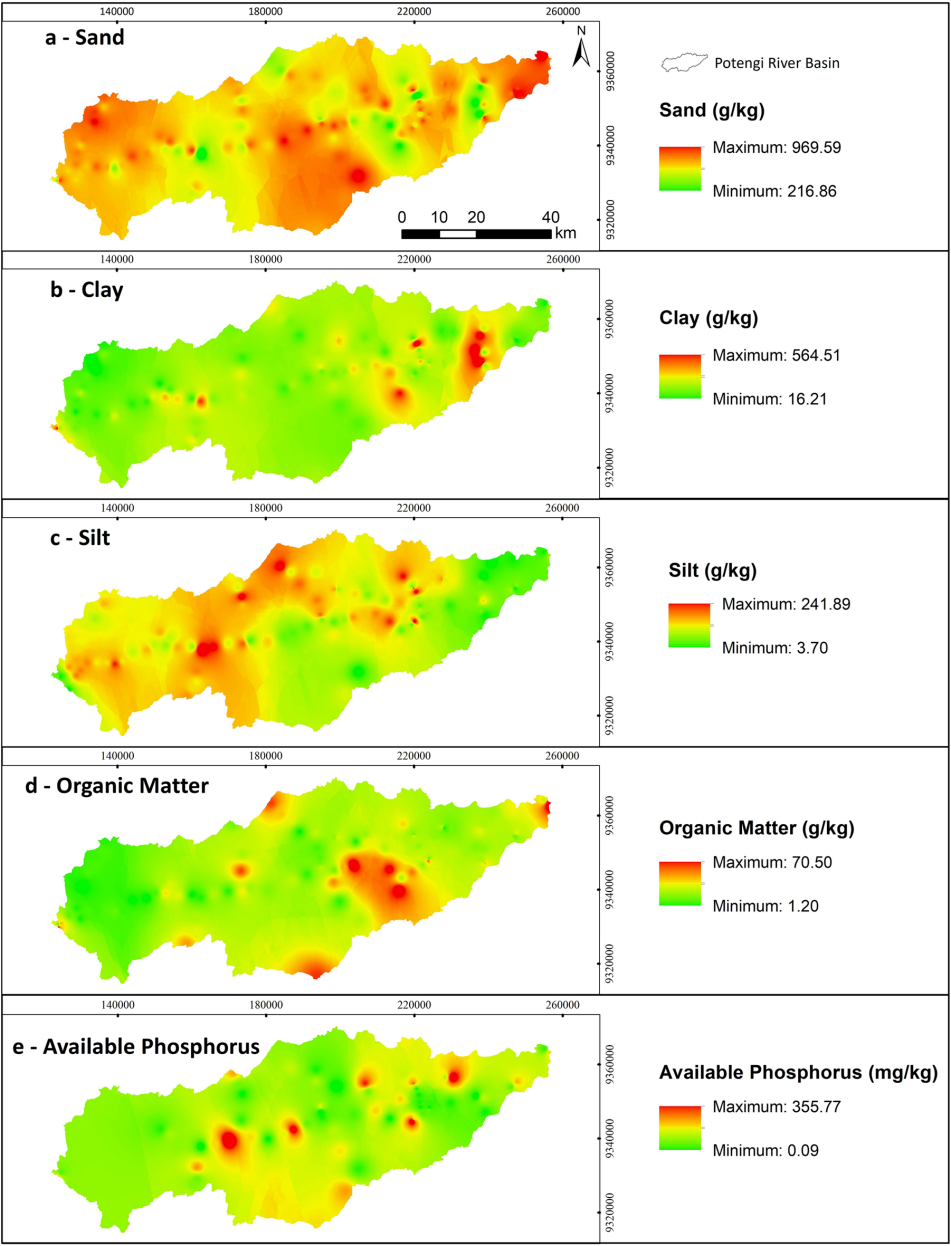
Table of maps of granulometric fractions, organic matter, and available phosphorus concentration in the soil. Sand (**a**). Clay (**b**). Silt (**c**). Organic Matter (**d**). Available Phosphorus (**e**). Source: authors (2025)

The spatial distribution of soil attributes in the PRB (Fig. 4) reveals a predominance of sandy soils across the basin (Table 1). The average sand content is 78.8%, ranging from 69.44% in Argissolo Vermelho Eutrófico to 89.06% in Gleissolo Tiomórfico Órtico. This predominance indicates that the soils have limited pollutant retention capacity and a high potential for diffuse pollution (Cunha et al., 2024). The elevated sand content also increases the basin’s vulnerability to erosion (Correa et al. 2016; Costa et al., 2018), as sand and silt particles aggregate less effectively than clay particles (Knapen et al., 2007).

**Table 1:** Physical and chemical properties of the soils of the Potengi River Basin

Types of soil	Sand	Silt	Clay	OM	P	Silt/clay	n^*^
	(g kg^−1^)	(g kg^−1^)	(g kg^−1^)	(g kg^−1^)	(mg kg^−1^)		
Latossolo Amarelo Distrófico	840.41^a^ ± 71.42^b^	35.85 ± 19.45	123.74 ± 65.45	21.23 ± 11.63	12.99 ± 14.54	0.29	21
	620.96^c^–921.63^d^	5.87–79.29	53.43–337.57	3.85–53.48	3.20–57.41		
Argissolo Amarelo Distrófico	712.36 ± 148.72	89.58 ± 51.64	198.06 ± 161.24	29.15 ± 19.55	77.17 ± 98.77	0.45	13
	389.97–865.41	34.89–207.65	50.25–564.63	5.25–61.53	1.18–338.13		
Argissolo Vermelho Eutrófico	694.40 ± 172.77	132.35 ± 46.40	173.25 ± 134.49	28.45 ± 17.94	47.43 ± 68.91	0.76	11
	216.85–878.82	61.57–221.55	56.70–561.60	8.72–70.47	0.10–204.22		
Neossolo Quartzarênico Órtico	839.82	19.68	140.5	27.49	84.82	0.14	1
Neossolo Litólico Eutrófico	796.81 ± 60.10	113.33 ± 35.96	89.86 ± 40.87	13.18 ± 9.11	109.78 ± 145.21	1.26	16
	674.19–910.54	67.96–202.65	21.50–166.07	3.86–37.20	3.11–323.54		
Luvissolo Crômico Órtico	790.13 ± 89.79	113.16 ± 48.21	96.72 ± 61.33	13.66 ± 12.32	45.13 ± 52.00	1.17	23
	478.09–888.75	18.21–241.91	26.60–280.00	1.15–46.50	0.02–152.27		
Planossolo Háplico Eutrófico	770.26 ± 60.87	113.47 ± 44.68	116.28 ± 41.29	20.05 ± 12.33	15.49 ± 15.29	0.98	12
	666.80–85.492	51.06–202.00	49.90–203.30	3.73–47.31	1.72–49.04		
Neossolo Flúvico Eutrófico	841.49 ± 95.55	80.84 ± 64.22	77.77 ± 50.60	14.85 ± 8.16	34.81 ± 21.23	1.04	7
	646.30–929.62	3.67–182.46	39.47–171.23	3.74–27.70	8.43–66.41		
Gleissolo Tiomórfico Órtico	890.59 ± 62.80	41.47 ± 22.84	67.94 ± 40.62	29.01 ± 23.66	116.42 ± 73.63	0.61	5
	830.20–969.64	14.16–62.45	16.20–111.95	10.33–70.21	5.48–209.90		
General	788.05 ± 110.28	91.43 ± 53.27	120.53 ± 90.65	19.36 ± 14.91	49.76 ± 72.72	0.76	-
	216.85–969.64	3.67–241.91	16.20–564.63	1.15–70.47	0.02–338.13		

Source: authors (2025)

^a^Average

^b^Standard deviation

^c^Minimum value

^d^Maximum value

^*^Number of samples

The mean values obtained for the different soil types are consistent with expected pedogenetic patterns. More developed soils, such as Latossolo Amarelo Distrófico, Argissolo Amarelo Distrófico, and Argissolo Vermelho Eutrófico, typically exhibit higher clay and organic matter content. In contrast, less developed soils, including Neossolo Flúvico Eutrófico, Neossolo Litólico Eutrófico, and Luvissolo Crômico Órtico, are generally characterized by higher sand content.

The average clay content in the PRB soils was 12.05%, ranging from 4.15% in the Gleissolo Tiomórfico Órtico to 19.81% in the Argissolo Amarelo Distrófico. Higher clay concentrations (Fig. 4b) were observed in localized areas, where enhanced water retention and nutrient adsorption capacities are expected. However, if these areas are poorly managed, they may also be more susceptible to compaction.

The average silt content in the soils was 9.14%, ranging from 0.37% in the Neossolo Flúvico Eutrófico to 24.19% in the Luvissolo Crômico Órtico (Fig. 4c). The spatial patterns of silt and clay correspond to soil classes characterized by an abrupt textural gradient between surface and subsurface horizons. This gradient results from the translocation and accumulation of clay and oxides, as observed in Argissolos, Planossolos, and Luvissolos. Notably, silt and clay content was negatively correlated with sand content (Table 2), indicating that areas with higher silt and clay concentrations are associated with superficial exposure of the B horizon, likely due to the erosive removal of the sandier A horizon.

**Table 2:** Pearson correlation coefficients for soil attributes. Source: authors (2025)

	Clay	Sand	Silt	Silt/clay	OM
Clay					
Sand	***r* = −0.78** ***p* = 0.013**				
Silt	*r*** = **0.15 *p* = 0.691	***r* = −0.74** ***p* = 0.023**			
Silt/Clay	*r*** = **−0.49 *p* = 0.182	*r*** = **−0.15 *p* = 0.696	***r* = 0.77** ***p* = 0.016**		
OM	*r*** = **0.47 *p* = 0.205	*r*** = **−0.12 *p* = 0.761	*r*** = **−0.32 *p* = 0.408	*r*** = **−0.64 *p* = 0.063	
P	***r* = −0.67** ***p* = 0.047**	*r* = 0.47 *p* = 0.205	*r*** = **−0.01 *p* = 0.974	*r*** = **0.35 *p* = 0.353	*r*** = **−0.04 *p* = 0.920

The values ​​in bold indicate the attributes that showed a significant correlation

The soils in the PRB have a silt-to-clay ratio ranging from 0.14 (Neossolo Quartzarênico Órtico) to 1.26 (Neossolo Litólico Eutrófico). This ratio is a reliable indicator of soil weathering, with values below 0.7 reflecting more advanced weathering. Four soil classes in the PRB: Argissolo Vermelho Eutrófico, Argissolo Amarelo Distrófico, Latossolo Amarelo Distrófico, and Neossolo Quartzarênico Órtico (Table 1), have ratios below 0.7. Despite the overall dominance of sand, the nine soil classes show distinct degrees of weathering. Notably, the Neossolo Quartzarênico Órtico, although composed of quartz-rich sand with low reactivity, can still undergo weathering, especially in tropical or semiarid regions where rainfall and humidity promote leaching. This process may transform primary minerals into secondary clay minerals, lowering the silt-to-clay ratio and making the soil more similar to highly weathered types, such as Latossolos or Argissolos.

The soils in the PRB exhibited moderate organic matter content (Fig. 4d), with an average of 19.36 g kg⁻^1^. The average values for each soil type did not exceed 29 g kg⁻^1^, which falls within the moderate range according to Prezotti and Garçoni (2013). This classification is based on concentrations between 15 g kg⁻^1^ and 30 g kg⁻^1^. Notably, the highest organic matter levels were found in more developed soils, such as Argissolos and Latossolos. These soils, characterized by greater structural stability and water retention capacity, may favor the accumulation of organic carbon over time.

The analysis of OM distribution in the soil shows a strong relationship with climate, weathering, land use, and management practices (Ramalho et al., 2025). In semiarid regions like the PRB, intermittent and intense rainfall events with high erosivity periodically remove the surface soil layer, promoting OM loss through water erosion. Additionally, high temperatures in these areas accelerate organic matter decomposition, resulting in rapid carbon cycling and increased mineralization (Araújo Filho et al., 2022).

The combination of these factors, along with degradation caused by agro-pastoral activities, may contribute to the reduced organic carbon content in the region’s soils, particularly in deforested areas and rainfed agriculture. These areas tend to have lower carbon stocks compared to areas with natural vegetation. In contrast, high OM levels (above 30 g kg⁻^1^) may be associated with irrigated agriculture, which can promote organic carbon accumulation due to increased biomass input and reduced mineralization. This is consistent with observations in the dry Caatinga soils, where irrigated agriculture has been shown to enhance organic carbon sequestration (Araújo Filho et al., 2022).

Furthermore, areas with higher organic matter content, especially in more weathered soils, also show increased phosphorus concentrations in their organic fractions. As soil develops, primary phosphorus is progressively weathered and incorporated into organic forms, even though the total phosphorus content tends to decline (Turner et al., 2007).

However, high organic matter levels reduce phosphorus adsorption and, consequently, increase its availability (Fink et al., 2016). This pattern highlights a strong connection between nutrient cycling and soil development within the watershed. As a result, organic matter dynamics not only affect soil fertility in the PRB but also play a key role in controlling phosphorus mobility and retention. This has important implications for the risk of eutrophication in regional water bodies, since phosphorus in organic-rich soils may be more easily mobilized.

According to CFSEMG (1989), P levels exceeding 35 mg kg⁻^1^ are considered high. Phosphorus availability in the soil reached levels of up to 338.11 mg kg⁻^1^ in the Argissolo Amarelo Distrófico (Table 1). While the highest individual phosphorus concentration was found in this soil, the highest average phosphorus levels by soil class were observed in the Neossolo Litólico Eutrófico and Gleissolo Tiomórfico Órtico.

However, this pattern is not fully reflected in the spatial distribution shown in Fig. 4e. The interpolated map highlights more pronounced phosphorus hotspots in the Luvissolo Crômico, followed by the Argissolo Amarelo Distrófico, which contains the sample with the highest P concentration. This apparent inconsistency underscores a limitation of the inverse distance weighting (IDW) interpolation method, which estimates values based solely on the spatial proximity of sampled points and does not account for soil class boundaries or pedogenetic variability. Consequently, areas with sparse sampling or high local variability may be smoothed or overrepresented, resulting in discrepancies between the mapped patterns and the tabulated data.

Recent studies evaluated the maximum phosphorus adsorption capacity (Smáx) of sandy soils in the PRB and other tropical regions, showing that Smáx values did not exceed 117 mg kg⁻^1^ in natural soils and 20.70 mg kg⁻^1^ in urban soils (Cunha et al., 2024). This indicates that phosphorus concentrations above Smáx cannot be retained by the soil, resulting in greater phosphorus mobility and increased risk of P loading in aquatic ecosystems.

The spatial distribution of phosphorus is partly explained by its significant inverse correlation with clay content (*r* = − 0.68, *p* < 0.05). Soils with higher clay content generally have lower extractable phosphorus, due to the strong adsorption capacity of clay minerals and iron and aluminum oxides (Jian et al., 2022), which promote phosphorus fixation. In contrast, sandy or low-clay soils, such as Neossolo and Gleissolo, have fewer sorption sites and thus tend to accumulate higher concentrations of labile phosphorus. This helps explain why high phosphorus levels occur in Neossolo and Gleissolo.

Although phosphorus transport mechanisms such as surface runoff, erosion, and leaching were not directly measured in this study, the evaluated soil properties are well established as key factors controlling these processes. Soil texture, OM, and P levels strongly influence P retention and determine the thresholds for P saturation and subsequent mobilization during rainfall events. Thus, integrating these attributes provides a reliable indication of the susceptibility of different soil classes to phosphorus loss under favorable hydrological conditions (Jovino et al., 2022).

The highest phosphorus concentrations were mainly observed in pasture and urban areas. This spatial pattern indicates inputs from external sources, such as intensive fertilizer use and organic waste disposal. Additionally, exposed soils and intensified agricultural activity near reservoirs promote the transport and mobilization of labile phosphorus, leading to its accumulation in sediments and increasing the risk of eutrophication in water bodies (Rocha Júnior et al., 2024).

In landscapes with intense or episodic rainfall, phosphorus loss is often linked to surface runoff and erosion, especially in soils with low clay content or poor aggregate stability. Under these conditions, phosphorus is mainly transported in particulate or weakly sorbed forms attached to fine sediments. Although this study did not measure phosphorus in sediments or runoff, the spatial variability of soil phosphorus and texture indicates that different soil classes have varying vulnerability, particularly in areas where there is surface connectivity between soils and drainage networks (Cunha & Cunha, 2023; Ramalho et al., 2025).

**Fig. 5:**
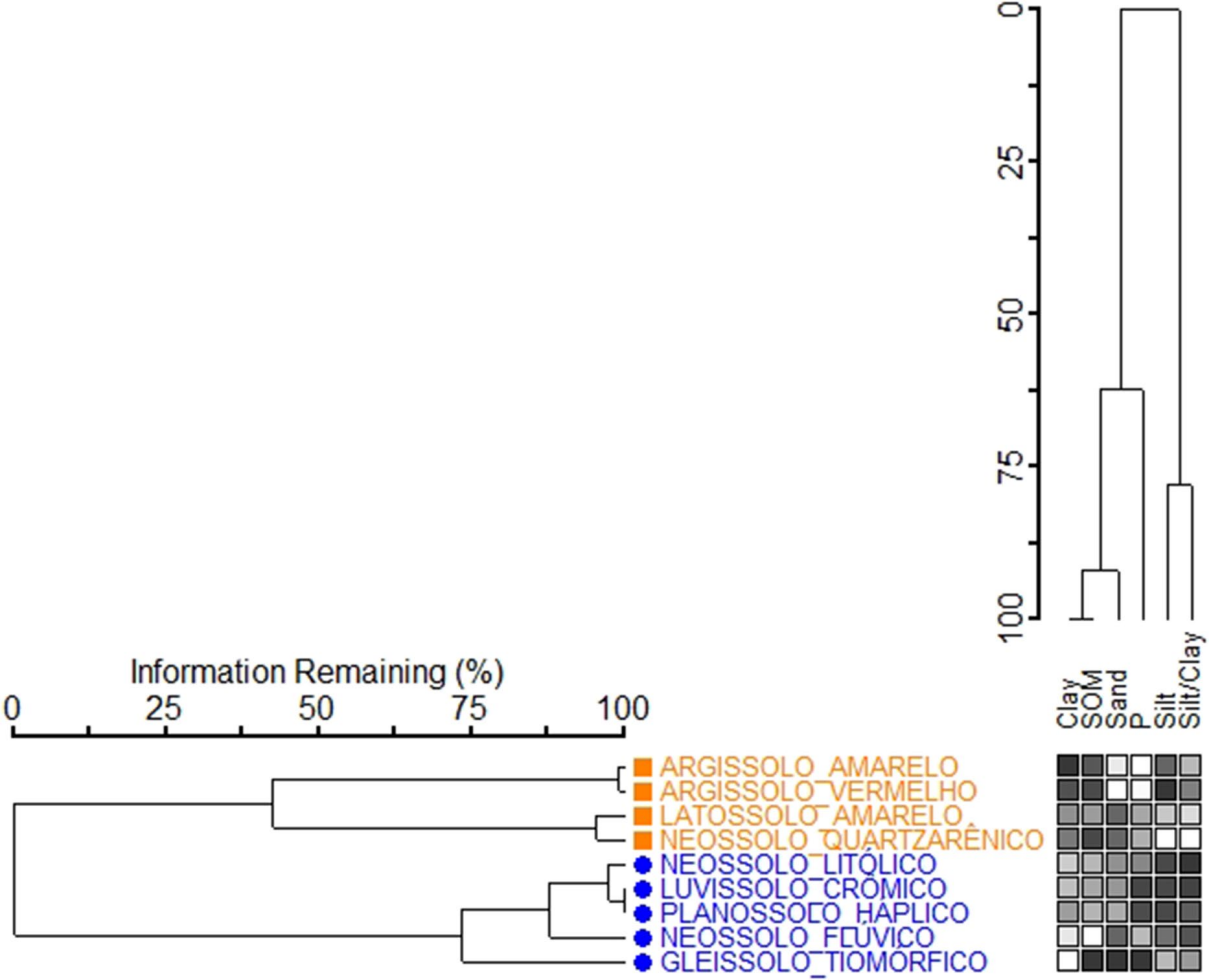
Two-way cluster analysis of soil attributes: more and less developed soil classes of the Potengi River Basin. Matrix: dark squares represent maximum values, while white squares represent minimum values for each property (columns) at sampling locations (rows). Source: authors (2025)

Two complementary statistical methods: two-way cluster analysis and PCA, were used to identify patterns and relationships among soil attributes across different soil types. The two-way cluster analysis revealed two distinct groups, corresponding to variations in soil properties and the degree of pedogenetic development (Fig. 5).

Group 1, comprising Latossolo Amarelo Distrófico, Argissolo Amarelo Distrófico, Argissolo Vermelho Eutrófico, and Neossolo Quartzarênico Órtico, includes four more developed soil types characterized by advanced weathering and predominantly well-drained conditions. In contrast, Group 2, comprising Neossolo Flúvico Eutrófico, Neossolo Litólico Eutrófico, Planossolo Háplico Eutrófico, Luvissolo Crômico Órtico, and Gleissolo Tiomórfico Órtico, encompasses five less developed soils, primarily associated with poor drainage and weaker pedogenetic differentiation.

**Fig. 6:**
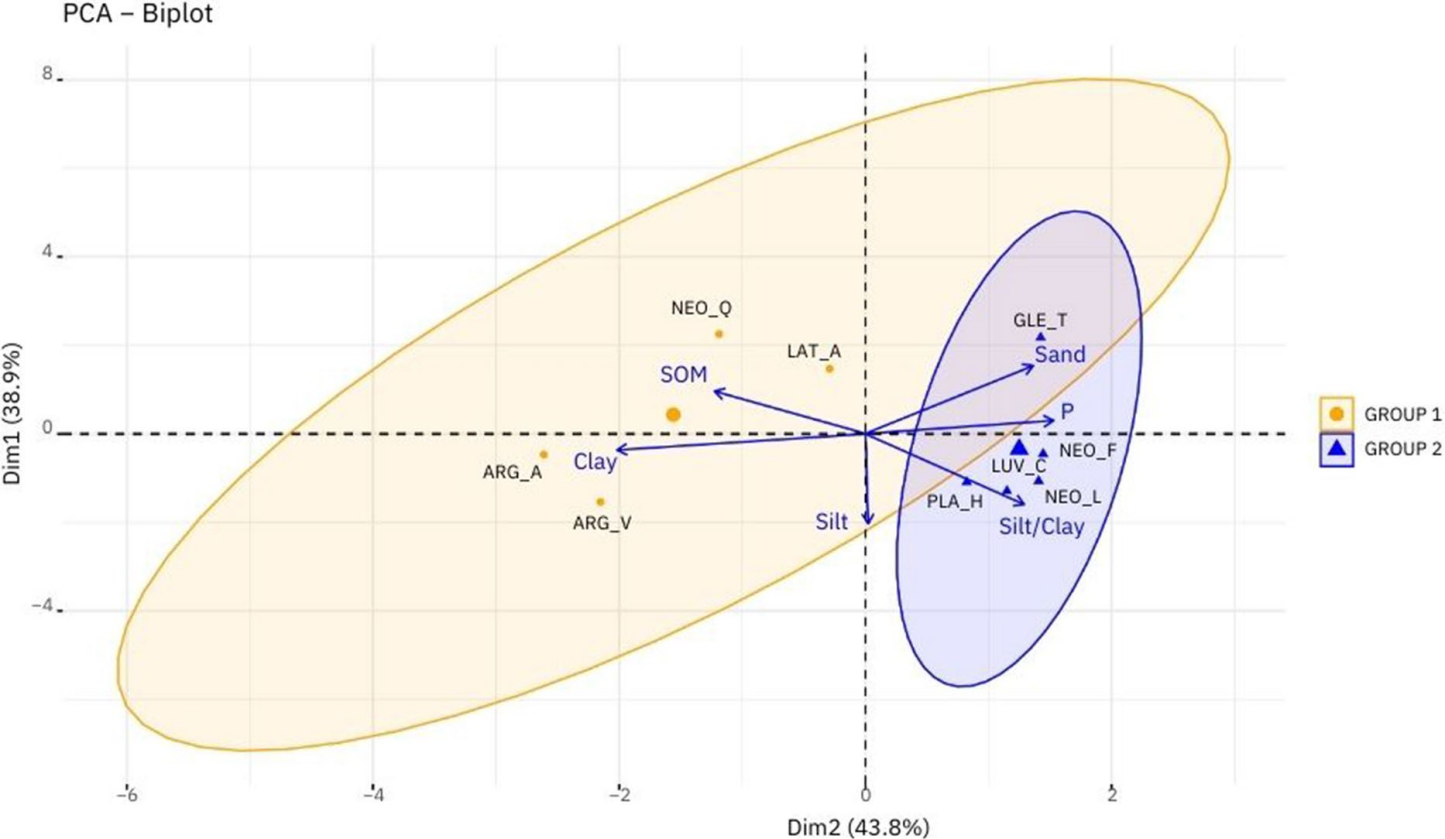
Principal component analysis (PCA) of six variables (physical and chemical attributes) in nine soil classes of the Potengi River Watershed in Rio Grande do Norte. S/A: silt-to-clay Ratio; Group 1: Latossolo Amarelo Distrófico (LAT_A); Argissolo Amarelo Distrófico (ARG_A); Argissolo Vermelho Eutrófico (ARG_V); Neossolo Quartzarênico Órtico (NEO_Q); Group 2: Neossolo Flúvico Eutrófico (NEO_F); Neossolo Litólico Eutrófico (NEO_L); Planossolo Háplico Eutrófico (PLA_H); Luvissolo Crômico Órtico (LUV_C); Gleissolo Tiomórfico Órtico (GLE_T). Source: authors (2025)

The PCA results confirmed the grouping pattern identified by the two-way cluster analysis and offered further insights into the variables responsible for soil differentiation. Using six variables, the PCA explained 82.70% of the data variability in the first two axes (Fig. 6). Clay, sand, organic matter, and phosphorus were the most influential variables on Axis 1, while silt and the silt-to-clay ratio primarily influenced Axis 2 (Table 3).

**Table 3:** Score matrix of the principal component analysis. Source: authors (2025)

	PC1		PC2
Clay	**−0.60**		−0.11
Sand	**0.40**		−0.20
Silt	0.01		**−0.64**
Silt/clay	0.38		**−0.50**
Organic matter	**−0.36**		0.30
Phosphorus	**0.45**		0.09
Standard deviation	1.62	1.53	
Proportion of variation (%)	0.44	0.39	
Cumulative proportion (%)	0.44	0.83	

The values ​​in bold indicate which axis each attribute has the greatest influence on

The PCA revealed a clear separation between the groups, with Axis 1 distinguishing four soil classes in Group 1 (characterized by negative scores) from five soil classes in Group 2 (characterized by positive scores). The more developed soils in Group 1 are strongly associated with higher clay and organic matter content, which are the main factors driving this classification. In contrast, the less developed soils in Group 2 have higher sand content, phosphorus levels, and silt-to-clay ratios, reflecting lower weathering degrees and a greater potential to contribute to diffuse phosphorus pollution in the watershed.

Assessing soil quality at the watershed scale is essential for effective environmental management, especially in regions at risk of phosphorus contamination that can degrade water quality. This study identified two distinct soil groups with different weathering degrees, with less developed soils posing a higher risk of phosphorus export to aquatic ecosystems. Such processes can intensify eutrophication, harming biodiversity and water uses. Watershed-scale analyses help pinpoint critical areas for monitoring and guide management practices to reduce nutrient transfer to water bodies, thereby supporting the sustainability of water resources and the conservation of aquatic ecosystems.

Although this study focused on a single watershed, its findings, especially regarding soil texture fractions, phosphorus, and organic matter content across soil classes, are consistent with international literature and reflect patterns typical of seasonally dry regions where semiarid and tropical climates coexist. In these landscapes, strong spatial heterogeneity in soil development, coupled with pronounced hydroclimatic seasonality and event-driven hydrology, concentrates nutrient mobilization into brief periods of intense surface runoff, sediment transport, and temporary hydrological connectivity between hillslopes and stream channels (Sun et al., 2025).

Given this broader context, the findings should not be generalized based on absolute phosphorus levels but rather on the underlying processes: (i) soil texture and organic matter as primary controls of phosphorus retention, desorption, and the size of labile phosphorus pools and (ii) the identification of soil classes more susceptible to phosphorus mobilization under hydrological connectivity. These relationships are consistent with global evidence that soil phosphorus status and physicochemical properties act as source controls, while hydrologically sensitive areas and event-driven transport processes determine the risk of off-site phosphorus transfer (Sun et al., 2025).

The finding that less weathered soils have higher extractable phosphorus concentrations, along with lower clay and organic matter content, is consistent with broader soil biogeochemistry evidence that texture and organic matter strongly influence phosphorus retention and mobility. In these less developed, coarser-textured soils with low OM, reduced adsorption capacity due to fewer clay mineral surfaces and weaker organo-mineral associations results in a greater proportion of phosphorus in labile pools, making it more susceptible to mobilization during periods of heavy rainfall (Baroutkoob et al., 2024; Jian et al., 2022).

This pattern has been observed in semiarid and seasonally dry landscapes, where topography, grazing intensity, and texture gradients influence the balance between phosphorus retention and its availability for lateral transport during heavy rainfall events (Sun et al., 2025). These mechanistic relationships indicate that less developed soils are inherently more vulnerable to phosphorus loss during episodic runoff. This concept can be cautiously extended to other seasonally dry regions with limited data, where similar soil and climatic controls prevail.

## Conclusion

- The soils of the PRB are predominantly sandy, with sand content exceeding 69% and clay content below 19% across all classes.
- Despite the sandy nature of these soils, the nine soil classes can be grouped into two distinct categories based on their degree of weathering.
- The distribution of silt and clay is closely tied to soils with an abrupt textural gradient, such as Argissolos, Planossolos, and Luvissolos, and can be influenced by erosion of the A horizon, leading to the superficialization of the B horizon.
- P in the PRB soils exhibits a significant inverse correlation with clay content (*r* = −0.68, *p* < 0.05), indicating that soils with higher clay content tend to have lower P.
- The low concentration of OM in some areas highlights the need for practices that promote soil quality improvement, such as organic fertilization and conservationist management systems.
- Less weathered soils with lower clay and organic matter contents (Group 2) tended to present higher extractable phosphorus concentrations, suggesting a greater potential for phosphorus to occur in more labile fractions. Under such conditions, the reduced retention capacity may increase the susceptibility of phosphorus to mobilization during intense rainfall events, reflecting pedogenetic controls commonly observed in semiarid and seasonally dry environments.
- The findings of this study contribute to a deeper understanding of the edaphic dynamics of the watershed and provide valuable insights for informing public policies aimed at sustainable natural resource management.
- Furthermore, this research emphasizes the importance of spatial approaches for monitoring soil quality and implementing effective management strategies to mitigate the impacts of diffuse phosphorus pollution on aquatic ecosystems.
- This study was based on soil samples collected from the 0–20-cm layer, which represents the most biologically active horizon and the portion of the soil most directly influenced by land use and management practices. However, this sampling strategy does not allow direct assessment of subsurface phosphorus dynamics, including vertical transport, leaching processes, or phosphorus accumulation in deeper soil layers. Consequently, the results should be interpreted as representative of surface soil conditions and as indicators of potential phosphorus availability and susceptibility to mobilization, rather than evidence of actual phosphorus fluxes to aquatic systems.
- This study recommends that further studies integrating soil, hydrological, and water quality data are needed to quantify the effective export of phosphorus and its implications for aquatic ecosystems.

## Data Availability

The data used in the research can be provided through a formal request.
